# Innovative moments in low-intensity, telephone-based cognitive-behavioral therapy for depression

**DOI:** 10.3389/fpsyg.2023.1165899

**Published:** 2023-07-25

**Authors:** Marie Drüge, Robert Staeck, Elisa Haller, Cara Seiler, Valentin Rohner, Birgit Watzke

**Affiliations:** ^1^Department of Psychology, Clinical Psychology with Focus on Psychotherapy Research, University of Zurich, Zurich, Switzerland; ^2^Faculty of Medicine, Institute of Social and Preventive Medicine, University of Bern, Bern, Switzerland; ^3^Faculty of Psychology, Clinical Psychology and Intervention Science, University of Basel, Basel, Switzerland

**Keywords:** depression, change process, innovative moments, reconceptualization, telephone-based cognitive-behavioral therapy, digital psychotherapy

## Abstract

**Background:**

Innovative moments (IMs), defined as moments in psychotherapy when patients’ problematic patterns change toward more elaborated and adaptive patterns, have been shown to be associated with a clinical change in patients with depression. Thus, far IMs have been studied in face-to-face settings but not in telephone-based cognitive-behavioral therapy (t-CBT). This study investigates whether IMs occur in t-CBT and examines the association between IMs and symptom improvement, and reconceptualization and symptom improvement.

**Methods:**

The therapy transcripts of *n* = 10 patients with mild to moderate depression (range: 7–11 sessions, in total 94 sessions) undergoing t-CBT were qualitatively and quantitatively analyzed. Symptom severity (Patient Health Questionnaire-9) and IMs (levels and proportions) were assessed for each therapy session. Hierarchical linear models were used to test the prediction models.

**Results:**

The rating of IMs was shown to be feasible and reliable using the Innovative Moments Coding System (IMCS) (84.04% agreement in words coded), which is indicative of the applicability of the concept of IMs in t-CBT. Only reconceptualization IMs were shown to have a predictive value for treatment success (*R*^2^ = 0.05, *p* = 0.01).

**Discussion:**

The results should be interpreted with caution due to the exploratory nature of this study. Due to the telephone setting, it was necessary to adapt the IMCS. Nonetheless, the extent of IMs identified in the low-intensity t-CBT investigated was comparable to IMs in face-to-face therapy. Further studies are needed to clarify the association between IMs and treatment success as a change process, especially for low-intensity treatments.

## 1. Introduction

Innovative moments are conceptualized as moments in psychotherapy in which the patients’ problematic patterns change toward more elaborated and adaptive ways of thinking, feeling, and acting and have, therefore, been discussed to predict symptom decrease and clinical change (Batista et al., 2020). To date, no study has identified IMs within a telephone-based cognitive-behavioral therapy for depression.

### 1.1. Innovative moments

Innovative moments (IMs) are behaviors, thoughts, or feelings that occur during the therapeutic dialog, which contrast the dominant and problematic self/life narrative (White and Epston, 1990). Although they occur in therapeutic dialog they can refer to past, present, or future (Gonçalves et al., 2012) and may occur spontaneously in psychotherapy sessions or may be prompted by therapists’ interventions. They can also occur between sessions (e.g., reflections on IMs between the session) and be addressed in the next session (Batista et al., 2020). Moreover, IMs can be understood as process measures (to activate change), and, therefore, be understood within the principles of change or outcome variables. Taking this into consideration, there are several other concepts such as the treatment and in-session processes (Kazantzis et al., 2018) or common factors of psychotherapy (Grawe, 2004), with which IMs are associated. However, investigating through the lens of IMs can provide more details about what is actually changing and what these changes entail through the different types and levels of IMs that can be identified. There are three types of IMs: action, reflection, protest, and reconceptualization, which can occur on three different levels. In level 1, IMs happen as “initial processes of changes,” in which client’s distance themselves from the maladaptive way of thinking or behaving, they may express new understandings of the problem, rejecting its assumptions or acting in a new way (Batista et al., 2020). In level 2 IMs, clients can tell what is changing (temporal contrasts to the maladaptive framework) or how/why it is changing (identification of the change process). In level 3 IMs, clients articulate both the changes in problematic behavior/self-narrative and their understanding of how this transformation is taking place, this is called “reconceptualization” (Batista et al., 2020). Therefore, IMs can be gateways for substantial therapeutic change (Gonçalves et al., 2009, 2017a) in the sense of weakening or even transforming problematic self-narratives. Even though IMs originate from a narrative tradition in psychotherapy, they can be identified across diverse therapeutic approaches (Gonçalves et al., 2021, see Table 1, column 2 for an overview). All therapies mentioned in Table 1 were on-site therapies.

**Table 1:** Study overview of innovative moments (IMs), therapeutic approach, disorder, interrater agreement, number of sessions, interrater reliability, and included clients.

	Therapy	IM-portion (%)	Sessions	Inter.-Rel. %, *κ*	*N* (Sessions)
Matos et al. (2009)	NT for victims of violence	GOG: *M* = 10.76 (SD = 4.84) POG: *M* = 5.38 (SD = 1.79)	*M* = 12.7 (SD = 3.74)	86 0.89	10 (127)
Mendes et al. (2010)	EFT for MD	GOG: *M* = 30.31 (SD = 4.02) POG: *M* = 8.90 (SD = 5.97)	*M* = 17.50 (SD = 1.87)	88.70 0.86	6 (105)
Gonçalves et al. (2012)	CCT for MD	GOG: *M* = 11.13 (SD = 5.50) POG: *M* = 5.82 (SD = 3.74)	*M* = 16.83 (SD = 0.98)	86 0.97	6 (93)
Alves et al. (2014)	Therapy for grief	*M* = 22.9	*M* = 13.83 (SD = 0.98)	86.45 0.86	6 (83)
Gonçalves et al. (2017b)	CBT for MD	GOG: *M* = 15.51 (RKS: 3.22) POG: *M =* 4.14 (RKS: 0.83)	*M* = 18.67 (SD = 3.27)	90 0.94	6 (111)
Gonçalves et al. (2017a)	NT for MD		*M* = 18.7 (SD = 1.83)	89.9 0.91	10 (180)

IM, innovative moment; Inter.-Rel., interrater reliability; *N*, sample; NT, narrative therapy; EFT, emotion-focused therapy; CCT, client-centered therapy; CBT, cognitive-behavioral therapy; GOG, good-outcome-Gruppe; POG, poor-outcome-Gruppe; RCS, reconceptualization; *M*, mean; SD, standard deviation; *κ*, Cohen’s Kappa for coded levels.

### 1.2. Telephone-based cognitive-behavioral therapy for depression

Depression is one of the most common mental health disorders, affecting approximately 280 million people worldwide (World Health Organization, 2021). However, there is insufficient use of healthcare services, especially for mild to moderate depression, which often does not correspond to the treatment guidelines as these suggest psychotherapy or a combination of psychotherapy and antidepressants rather than antidepressants alone (World Health Organization, 2021). Telephone-based cognitive-behavioral therapy (t-CBT) attempts to address potential barriers to treatment (e.g., going to the clinic on-site, shame). Some t-CBT can be classified as guided self-help (although in a rather intensive form), whereas in other t-CBTs only the setting differs from face-to-face CBT. In a meta-analysis including 12 trials by Mohr et al. (2008), a significant pre–post improvement of depressive symptoms (*d* = 0.81, *p* < 0.0001) and a significant superiority of telephone psychotherapy over control groups including treatment-as-usual or minimal intervention (*d* = 0.26, *p* < 0.0001) was found. Beyond symptom improvement, the rate of therapy dropout of 7.6% was lower in the telephone setting than for on-site treatments. In a randomized-controlled trial (Mohr et al., 2012), in which 325 participants with depression were treated with 18 sessions of CBT, the reduction in symptoms did not differ between the two conditions (telephone vs. on-site). However, dropouts in the t-CBT group were significantly lower than in the on-site treatment (20.9% vs. 53%). In their meta-analysis including 10 randomized-controlled trials, Castro et al. (2020) found significant symptom improvement with t-CBT in the pre–post comparisons. Although digitalized psychotherapy process research holds the potential to enhance process research due to specific properties such as ecological momentary assessment, is a rather novel branch of research (Domhardt et al., 2021). In a narrative review by Berger (2017), therapeutic alliance in Internet interventions (e.g., real-time video-conferencing therapies, e-mail therapies, and chat therapies) is described as equivalently rated compared to face-to-face therapies independent of communication modalities, diagnostic groups, and amount of contact, thereby suggesting that a positive alliance can be established in Internet interventions (Berger, 2017). The review also provides an overview of alliance-treatment outcome associations and concludes that the affective bond between the patient and therapist might be less important in Internet interventions than in face-to-face therapy as none of the studies provided evidence for an association between the personal bond and treatment outcome (Berger, 2017). Findings from qualitative research also indicate how a positive therapeutic alliance can be fostered in Internet-based CBT by focusing on the four basic needs (e.g., attachment) through certain therapeutic techniques (e.g., active listening and validation) (Theurer and Wilz, 2023). However, little is known about whether similar processes occur in telephone-based psychotherapy (i.e., t-CBT) as in on-site psychotherapy.

### 1.3. Innovative moments and depression

The concept of IMs has been investigated in several process–outcome studies; however, future studies must expand our knowledge by employing disentangling research questions and appropriate study designs. Overall, the main findings are that effective psychotherapies differ from less effective ones by (a) a higher overall percentage of IMs and (b) specifically by a higher percentage of level 3 IMs (Gonçalves et al., 2017a). In contrast, no significant difference in the aforementioned groups resulted so far in the occurrence of IMs of low levels—regardless of the therapeutic approach (Gonçalves et al., 2017a). As shown in Table 1, IMs have been studied several times in clients with depression, although not in digitalized psychotherapy such as t-CBT. IMs were identified as reliable predictors of depressive symptomatology in CBT (Gonçalves et al., 2017b). Gonçalves et al. (2017b) confirmed a reliable use of the IMCS in CBT (percent agreement in the numbers of coded IMs words: 90%, Cohen’s Kappa regarding IMs levels: 0.94) on six clients who had been diagnosed according to the Diagnostic and Statistical Manual of Mental Disorders (DSM-IV). To compare depressive symptomatology, two groups of three individuals each were formed, one group responded to treatment and the other one did not. The former showed a mean IMs percentage of 24.14% with 3.22% level 3 IMs, while the latter had a mean IMs percentage of 15.51% with 0.83% level 3 IMs. In a single-case study that was derived from the trial, Fernández-Navarro et al. (2018) examined the treatment transcript of a 39-year-old Portuguese woman who suffered from major depression. After completing narrative therapy, the patient was considered remitted. Interestingly, she showed the highest proportion of level 3 IMs compared to the rest of the participants. In another study, two samples were merged resulting in a total contingent of 7,903 level 3 IMs (with a mean salience of 1.95%, SD = 2.18) (Fernández-Navarro et al., 2018), which were further used as predictors of symptom change. According to Fernández-Navarro et al. (2018), all three integrated predictors (level 3 IM, contrasting self: what has changed and change process: how it changed) were significantly predictive of symptom improvement in each subsequent therapy session, provided that a separate model was calculated for each of the three level 3-IM predictors: *R*^2^*corr*. = 0.59, contrast: *R*^2^*corr* = 0.59, change process: *R*^2^*corr* = 0.58). When all three variables were integrated into the same hierarchical–linear model, only level 3 IMs showed significant predictive performance (*R*^2^*corr* = 0.60). However, these results should be interpreted with caution due to the study design and as the first indications that IMs and therapy outcomes in depression may be related.

To date, IMs have been studied in face-to-face psychotherapy but never in t-CBT, therefore, this study pursues the following objectives:

1. Investigating IMs in a t-CBT.
2. Predicting depressive symptoms post t-CBT with IMs.
  2.1. How is the total percentage of IMs associated with the depression score at the end of therapy compared to the beginning of therapy?
  2.2. How are level 3 is IMs in one therapy session associated with a decrease in depressive symptoms in the next session?

## 2. Methods

### 2.1. Study design

The present study applies a descriptive, explorative mixed-method design. Therapy transcripts were coded qualitatively using the innovative moments coding system and then analyzed quantitatively. For this purpose, a secondary analysis using a correlative design was carried out on a sample, that is, based on a randomized-controlled trial (Watzke et al., 2017). The data in the present study were composed of therapy transcripts (for further information: Haller and Watzke, 2021) of 10 patients (94 sessions in total) who were part of a larger study aimed to analyze the effectiveness of a telephone-based cognitive-behavioral therapy (Watzke et al., 2017) as well as homework engagement (Haller and Watzke, 2021).

#### 2.1.1. Patients

For the present study, data from the intervention group have been used (*N* = 24). A sufficient proportion (>80%) of the therapy sessions was available in transcribed form for 21 of the participants, as some recordings were missing due to technical reasons. For this study, 10 patients were selected based on their improvements (pre–post-treatment) on the PHQ-9, which is in line with previous studies applying a similar procedure (e.g., Batista et al., 2020), as this method includes different changes in symptomatology. Of the 10 selected patients, seven were female subjects. At baseline, 10 subjects were 59.9 years old on average (SD = 18.2, range: 25–79) and had an average PHQ-9 score of 13.4 (SD = 4.6, range 6–20) indicating a moderately depressed sample (Kroenke and Spitzer, 2002). The baseline PHQ-9 scores were compared with the scores at the end of the therapy, and patients with different levels of decrease in symptomatology in PHQ-9 (pre–post: −11, −11, −8, −6, −6, −5, −3, −2, 0, +2) were selected. The patient characteristics are described in the results section (see Table 2 for more details).

**Table 2:** Innovative moments in telephone-delivered cognitive-behavioral therapy are divided into three levels and seven types.

Levels	Types and definition	Contents (examples)	Examples in t-CBT
Level 1 Centered on distancing from the problem (low level) Allow the detachment from the problematic experience using moments critique, needs, doubts, coping-strategies	Action I: Performed or intended behaviors	• New behavioral strategies to overcome the problem(s) • Active exploration of solutions • Searching for information about the problem(s)	*Action I: “And […] the feeling that I described before, which I felt over the course of the afternoon, I have only in the course of the evening then so in connection with uh with the togetherness with the friends, with the common meal and the common conversations actually dissolved.”*
	Reflection I: New understandings of the problematic experience and its effects	• Reconsidering the causes of the problem(s) • Awareness of the effects of the problem(s) • Formulations of new problem(s) • Adaptive self-instructions and thoughts • Intention to fight demands of the problem(s) • General references of self-worth and/or feelings of well-being	
	Protest I: Objecting the problem and its assumptions	• Rejecting problem(s) or objecting to the problem(s) • Position of critique towards others who support it • Position of critique towards problematic facets of oneself	
Level 2 Centered on the elaboration of change (high level) New aims, experiences, activities or projects, anticipated or in action, as a consequence of change (not directly related to the problematic experience)	Action II: Generalization of good outcomes into the future and other life dimensions	• Investment in new projects or relationships as a result of the process of change • New skills unrelated to the problem • Problematic experience as a resource for new situations	*Reflection II: “And then we came to the common theme of learning to accept what is, and how difficult that is in everyday life, of course. Because we are both more movement and sport-oriented, actually we both liked the dynamic life somehow. And now we, he even more than I, are somehow confronted with this and it did us a lot of good to talk about it again with a friend who knows about it from his own experience.”* *Action II: “So (um) what I take with me are the (um) keeping to the daily structure, and simply paying more attention and also enjoying the beneficial activities. And simply um that it is also um yes that it is that that it is important that I am also aware of this. And not to take it for granted. That it is like that and that I can actually experience a relatively rich activity every day, actually. Or that is (um) starting with the housing situation, and all the things that I can still do myself. Actually, um that’s actually very very very much. And (um), and I just take that with me and try to make myself aware of it again and again.”*
	Reflection II: Elaborations upon change and its consequences	• What is changing • Generating meaning/insight about how/why changes are occurring • References of self-worth and/or feelings of well-being (as consequences of change)	
	Protest II: Assertiveness and empowerment	• Centering on the self • Affirming rights and needs	
Level 3 Integration of new meanings in an articulated way	Reconceptualization: Meta-cognitive process description; articulates a shift between two self-positions and access to the process underlying this transformation	Contrasting self (what changed/is changing?) AND Change process (how/why change occurred/is occurring?)	*Reconceptualization: “Client: I really did, I think I did it well now. So now there is simply a new side to me.* *Therapist: What helped you to master this situation?* *Client: To have the inner certainty that I know that the truth, that is, I had an inner truth where I said, even if it says the opposite, I know what is and what I actually need. And I do not want to back down. I actually have no reason not to appear.”*

Adapted from Gonçalves et al. (2019) and Batista et al. (2020).

#### 2.1.2. Therapists

Three therapists from the Psychotherapy Outpatient Center of the University of Zurich conducted the t-CBT. On average, the three therapists were 34 years old (SD = 5.9), in advanced, postgraduate training to become CBT therapists (average duration of training: *M* = 4.3 years, SD = 1.5) and had experience in treating patients with depression. Before the start of the study, they were trained by an experienced clinical psychotherapist and researcher in t-CBT and were supervised regularly during the study.

### 2.2. Treatment

The patients in the intervention group first met face-to-face with their assigned therapist, followed by 8–12 telephone sessions as digital remote treatment. In the beginning, sessions took place weekly, later, and by arrangement fortnightly, and lasted approximately 40 min on average. The treatment was structured along the manualized guided self-help CBT “creating a balance” (Simon et al., 2004; Steinmann et al., 2016). The program, designed as a low-intensity, short-term intervention, was based on a therapist manual and a client workbook. The patients were asked to use a workbook in between the sessions. The content of the therapy comprises psychoeducation, activity-building activation, cognitive restructuring and self-control, and relapse prevention. Therefore, it is a rather intense form of guided self-help with approximately 450 min of human interaction.

### 2.3. Measures

#### 2.3.1. Patient Health Questionnaire-9

Depressive symptoms were assessed by the German version of the Patient Health Questionnaire 9 PHQ-9 (Löwe et al., 2002) and rated on a 4-point Likert scale with values between 0 = “not at all” and 3 = “nearly every day” at the beginning of each session. The final score is calculated as the sum of all items. Kroenke and Spitzer (2002) defined the following cutoff values of the PHQ-9 regarding the severity of a depressive episode: 0–4 points = no depression; 5–9 points = mild; 10–14 points = moderate; 15–19 points = moderate to severe; 20–27 points = severe. Studies provide evidence that the PHQ-9 has satisfactory psychometric properties (internal consistency: 0.82) if used on the phone (Pinto-Meza et al., 2005).

#### 2.3.2. Innovative moments coding system

The innovative moments coding system (IMCS) (Gonçalves et al., 2011, 2019) proposes a systematic way of tracking the transformation of clients’ maladaptive framework of meanings through the identification of IMs in transcripts or videos of psychotherapeutic sessions. In most studies, two raters perform the coding independently, one codes 100% of the material, and the second one codes between 30% and 100%. Afterward, the interrater agreement indices are calculated according to the proportion of IMs words in transcripts/proportion of IMs time in video ratings. An intercoder agreement between 84% and 94% (Gonçalves et al., 2011) has been accepted as reliable, and the interrater reliability of the coded levels is reported as acceptable between 0.80 and 0.97 (Gonçalves et al., 2011). See Table 1 for an overview of intercoder agreement (column 3) and reliability (column 5).

#### 2.3.3. Innovative moment coding system for t-CBT

The IMCS allows for the identification of three different levels of IMs. The interrater reliability of the IMCS has been demonstrated in the context of different disorders and therapeutic approaches (Gonçalves et al., 2011). The average agreement on coded words in previous studies ranged between 84% and 94%, the Cohen’s Kappa for the coded types and levels between 0.80 and 0.97, which indicates an adequate interrater agreement (Hill and Lambert, 2004, see Table 1 for IMCS). As the IMCS has not been applied in t-CBT before, certain assumptions and adaptations were made due to the communication *via* telephone (see Supplementary material I). The current manual of the IMCS (Gonçalves et al., 2019) was used to identify IMs and rate their level. The two raters were trained in a 5-step training guided by two experienced coders for several weeks on standardized material first (see Batista et al., 2020 for more detail) and then on the material of this study. Based on the first transcript of each session, the coders created a problem list derived from what the patient had said within the first session and continuously updated this problem list throughout the therapy for each patient which resulted in covering central problem areas. The raters met regularly during the analysis process for interactive and collaborative discussion of the sessions analyzed by both raters for final consensus-based coding. Based on the first coding, interrater reliability was calculated, and for the analysis, consensus-based coding was used. Out of the 94 therapy transcripts, 75% were coded by both raters. The two raters matched 84.04% of the words identified as IMs (in 75% of the sessions both coded). The Cohen’s Kappa for agreement on IMs levels was 0.93, which is above the minimum value of 0.75 required by Gonçalves et al. (2019) and corresponds to a high level of agreement according to Hill and Lambert (2004).

### 2.4. Statistics

All calculations and all graphs illustrating the results were carried out and produced using the statistical program R (R Core Team, 2019). Interrater reliability was assessed to capture the extent of a reliable application of the IMCS in t-CBT. For this purpose, the percentage agreement was calculated with regard to the number of words that had been classified as IMs, first for each session and later for all words spoken in all therapies. The words assessed as IMs by both raters were divided by the total number of IMs words identified. Furthermore, the agreement between both raters on the levels was assessed by determining Cohen’s Kappa. A linear regression was computed to investigate the association between IMs and treatment response. Additionally, single regression models were calculated by including each level as a predictor separately. Hierarchical linear models (HLMs) were computed to calculate whether the number of IMs in one session was predictive of a decline in depressive symptomatology in the subsequent session. HLM was performed using non-linear mixed-effect modeling with fixed effects using the R package lme4 (Bates et al., 2014). According to the QQ-plot of the residuals, the error values were both normally distributed and on average zero, the prerequisites for the application of the HLM were fulfilled accordingly.

## 3. Results

### 3.1. Innovative moments in telephone-based CBT

Innovative moments (IMs) were found in t-CBT for depression. Table 2 provides an overview of the levels and types of IMs along with their contents and examples in t-CBT.

In sum, 1,129 IMs were coded throughout all therapy transcripts (see Table 3 for more detail on IMs for each patient). Level 1 IMs occurred 973 times and comprised on average 6.77% of all the words spoken in all sessions (SD = 2.73%). A total of 175 IMs were coded as level 2 IMs (2.98% of all the words spoken in all sessions (SD = 2.32%)) and 17 as level 3 IMs (0.64% of all the words spoken in all sessions (SD = 0.93%)). The average percentage of IMs per session was 10.39% (SD = 4.99%). While IMs of level 1 and level 2 were found in all therapies, no level 3 IMs were found in four patients. As the level increases, the average number of words per IMs approximately doubled (level 1: 51 (SD = 51), level 2: 101 (129), level 3: 213 (141)). The number of words per IMs differed significantly (*p* < 0.01) between the three levels.

**Table 3:** Innovative moments and depressive symptoms for each patient.

		PHQ-9		Innovative moments (%)			
ID	*N*_s_	Pre	Post	Level 1	Level 2	Level 3	Total
1	7	11	0	4.87	2.39	0.35	7.61
2	10	7	7	6.61	1.19	0	7.8
3	11	18	12	7.48	1.96	0	9.44
4	10	14	12	3.22	0.71	0	3.93
5	9	9	4	12.15	7.76	0	19.91
6	8	9	3	7.61	3.28	0.71	11.61
7	9	12	1	7.44	4.73	1.99	14.16
8	9	17	9	6.31	5.42	2.6	14.32
9	11	6	8	9.07	1.71	0.62	11.4
10	10	9	6	2.96	0.68	0.09	3.72
M (SD)	9.4 (1.26)	11.2 (4.05)	6.2 (4.21)	6.77 (2.73)	2.98 (2.32)	0.64 (1.00)	10.39 (4.99)
Average (words per IM)				51 (51)	101 (129)	213 (141)	

ID, participant identification code; *N*_s_, number of therapy sessions; PHQ-9, Patient Health Questionnaire-9 items; Pre, pre-therapy; Post, post-therapy.

**Figure 1:**
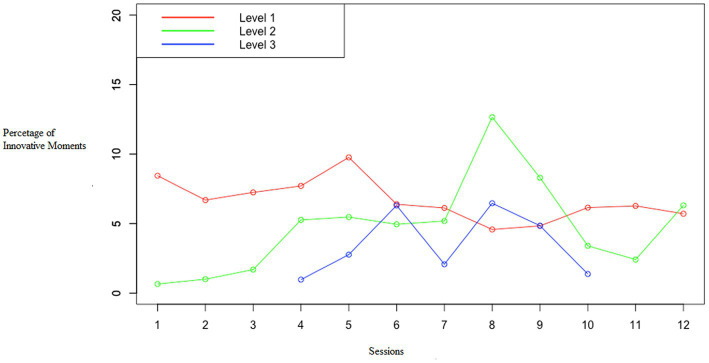
Level-specific percentages of IMs for therapy.

The percentage of different level IMs varied throughout the sessions (see Figure 1, individual courses of level-specific percentages of IMs and depression can be found in Supplementary material II). The individual depression and IMs trajectories in Supplementary material II show that L1 and L2 IMs were found in all patients over the entire duration of therapy. In contrast to L2 IMs, which did not occur at all or only sparsely in most of the patients at the beginning of therapy and in eight out of 10 in the first therapy session, all patients except for patient 5 (in session 7) consistently showed L1 IMs. While four of the 10 patients did not show a single L3 IMs throughout the entire therapy, L3 IMs tended to appear in the second half of the therapy for the other six patients, earliest from session 4 onward. For the patients, in which L3 IMs were found (patients 1, 7, 8, and 10), a parallel change in depressive symptoms can be seen in both directions, all of them were patients with the strongest decrease in depressive symptomatology.

### 3.2. Association of IMs with treatment outcome

The overall number of IMs was not significantly associated with *p* = 0.42 with a decline in depression (pre–post-treatment, 𝛽 = −0.25, 𝑅^2^ = 0.08, *p* = 0.42). Testing the levels separately also revealed that there was no significant association between any level and the decline of depression. Results of the linear regressions can be found in Supplementary material III. Subsequently, an HLM was calculated with L3 IMs as a predictor and the decline of symptoms in the following session as the outcome. L3 IMs in one therapy session significantly (𝛽 = −0.25, *𝑅*^2^ = 0.05, *p* = 0.01) predicted a decrease in depressive symptoms in the next session.

## 4. Discussion

This is the first exploratory study focusing on IMs in the context of a telephone-delivered psychological treatment following the principles of a CBT approach to depression, i.e., in the context of low-intensity and remote treatment. Some adaptations had to be made due to the telephone setting, e.g., reassuring a stable connection or how to code answers of clients to PHQ-9-monitoring due to the specific intervention program and the telephone setting (see Supplementary material I for more detail). Overall, IMs were detectable in t-CBT; therefore, the IMCS was found to be feasible for transcripts of telephone-delivered therapy sessions. Moreover, we found high interrater reliabilities, comparable to those in face-to-face psychotherapy (see Table 1, Gonçalves et al., 2021). This is the first study examining whether the same change processes found in face-to-face therapy also emerge in a remote format of treatment. Interestingly, the average proportion of words classified as IMs (6.77%, SD = 2.73%) per session and the average proportion of IMs in each session (10.39%, SD = 4.99%) are comparable to other studies (see Table 1, e.g., Gonçalves et al., 2012). While IMs of level 1 and level 2 were found in all therapies of our patient sample, level 3 IMs were not found in four patients. Due to the content and format of the treatment manual used, it is unsurprising, that most of the IMs found in t-CBT were level 1 or level 2 as the treatment is designed as a short-term intervention focusing on core elements of CBT for depression, e.g., behavioral activation, bringing actions into everyday life (most likely L1 IMs or L2 IMs, see Table 2). Given the brevity of the manual, integrating new meanings of self-narratives was not emphasized (except cognitive reconstruction). Interestingly, level 3 IMs were still found in six patients, who showed a pronounced symptom improvement, which may have been stimulated by cognitive restructuring. The extent to which short-term interventions (i.e., minimal interventions and e-mental health interventions) can also stimulate reconceptualization processes (L3 IMs) and how this is related to symptom improvement should be the subject of further research. However, whether IMs are predictors or outcomes of therapy remains in question.

A major strength of this study is that all sessions were coded rather than a pre-defined selection of sessions (e.g., first, fifth, and last session of each case) being coded. Therefore, the change in IMs level and extent of IMs can be seen as a continuous process rather than only in a limited selection of therapy phases. This is advantageous because the occurrence of IMs in one session can be directly linked to current symptom severity and its process. Interestingly, the individual trajectories (IMs and PHQ-9) show a wide range of changes in symptomatology and IMs. In this context, it would be interesting to identify the moments that could have fostered L3 IMs. Both intrasession and intersession processes may have to be considered.

Our findings are in line with prior results regarding the association between depressive symptoms and IMs, thus there is no causal interpretation, but a prediction of decreases in depressive symptoms from increases in L3 IMs. Nevertheless, some limitations need to be considered: Despite the high number of coded sessions (96), it is a small sample of 10 participants. Therefore, the results of this exploratory study need to be interpreted as such and with the utmost caution; studies with larger samples are needed. In addition, and in line with previous research, 10 patients with different extents of symptom changes between pre- and post-treatment were selected (e.g., Batista et al., 2020), which may have artificially increased the variance and should be reconsidered for studies with larger samples including the full range of symptom change.

The assessment of depression over the telephone might also have led to effects of social desirability; however, research has proven the feasibility of PHQ-9 over the telephone (Pinto-Meza et al., 2005). However, a more specific measure of depression could address this problem through an ecological momentary assessment. Additionally, blinding of coders was not possible because the PHQ-9 was a part of the beginning of each session and could not be cut out of the audio recording as it involved relevant information due to more detailed patient responses. Therefore, the coders were aware of the patient’s current symptom severity. This may have led to an overestimation of the level of IMs in situations when patients expressed less burden. However, how much the raters actually payed paid attention to the change in PHQ-9 remains in question as they were focused on coding IMs. Interestingly, the IMs found were often in the context of reflection on homework and moments of change, which represented a large part of this format of t-CBT (Haller and Watzke, 2021). Therefore, the overlap between homework engagement and IMs remains unclear in this specific intervention and could be addressed in further research. Perhaps a systematic analysis of the types of IMs (e.g., type “action” during behavioral activation) or qualitative content analysis (are there other moments of relevance/change) could also help to clarify the IMs of relevant change in this specific intervention. As in our results, level 3 IMs have been of particular interest in previous research (Gonçalves et al., 2017a; Fernández-Navarro et al., 2018), as they have been found to be predictive of a decrease in depressive symptoms. This may lead to a clinical and research interest: How could level 3 IMs be promoted by the therapist? Are there specific methods or strategies to promote level 3 IMs, i.e., reflective questions on change processes? Educating psychotherapists in the IMs concept and especially sharpening their focus to level 3 IMs could be addressed in clinical practice.

Nonetheless, this study can be seen as an approach to identifying a process of change in t-CBT for depression that was first found in face-to-face therapy. In order to justify the derivation of practical implications, testing for a causal relationship between IMs and treatment success as a change process still remains, especially for low-intensity treatments.

## Data availability statement

The data analyzed in this study is subject to the following licenses/restrictions: The raw data supporting the conclusions of this article will be made available by the authors, without undue reservation. Requests to access these datasets should be directed to marie.druege@uzh.ch.

## Ethics statement

This study was performed in line with the principles of the Declaration of Helsinki and was approved by the local Ethics Committee of the Canton of Zurich (Ref. Nr. 2015-0417). The patients/participants provided their written informed consent to participate in this study.

## Author contributions

MD and RS planned and conceptualized the study, and trained and supervised CS and VR to collect, analyze, and interpret the data. MD drafted and revised the manuscript. EH supervised the transcription process and revised the manuscript. BW supervised the study, involved in the study conceptualization and interpretation of data, and revised the manuscript. All authors contributed to the article and approved the submitted version.

## Conflict of interest

The authors declare that the research was conducted in the absence of any commercial or financial relationships that could be construed as a potential conflict of interest.

## Publisher’s note

All claims expressed in this article are solely those of the authors and do not necessarily represent those of their affiliated organizations, or those of the publisher, the editors and the reviewers. Any product that may be evaluated in this article, or claim that may be made by its manufacturer, is not guaranteed or endorsed by the publisher.
