# Supplementary material for: Innovative moments in low-intensity, telephone-based cognitive-behavioral therapy for depression
Source: Front Psychol. 2023 Jul 25;14:1165899. doi: 10.3389/fpsyg.2023.1165899 (PMC10409642; doi:10.3389/fpsyg.2023.1165899)
Supplement: Supplementary file 1 [file Data_Sheet_1.docx]

Supplementary material I

**Coding Rules Innovative Moments Coding TiDe**

P=patient, T=therapist, Innovative Moments

Basic

1. An IM is starts and ends with P.

2. If IMs occuring repeatedly, they are coded. Repeated IMs are coded again.

3. Coding is close to the text (more distanced from assumptions, interpretations).

4. If in doubt, code an IM rather than not. We will discuss

5. A reflection process can be started/prompted by the T in therapy, the IM in this case starts with P's answer. Such an IM is online (still debatable if necessary).

6. Ambivalences end/interrupt IMs, so they are not counted in the wordcount.

7. Statements by T between two statements by P are counted if both statements by P are IMs.

8. Screening answers (PHQ-9) are not IMs per se, unless P elaborates on the train of thought or gives examples.

9. Pure affirmation/denial by P of T's suggestions is not yet an IM (except as part of a longer IM in the middle), a process of reflection/processing must be recognisable (e.g. through extensions, examples, concretisations on the part of P).

Level

10. Plans for individual actions/or individual actions carried out between two sessions are Level 1 (Action 1).

11) Individual actions carried out with longer-term effects (e.g. divorce, possibly dyed pink hair?) are Level 2. In addition, everything where a pattern is broken with a more permanent character (Contrasting Self or Change Process).

12. As soon as Change Process and Contrasting Self occur together, level 3.


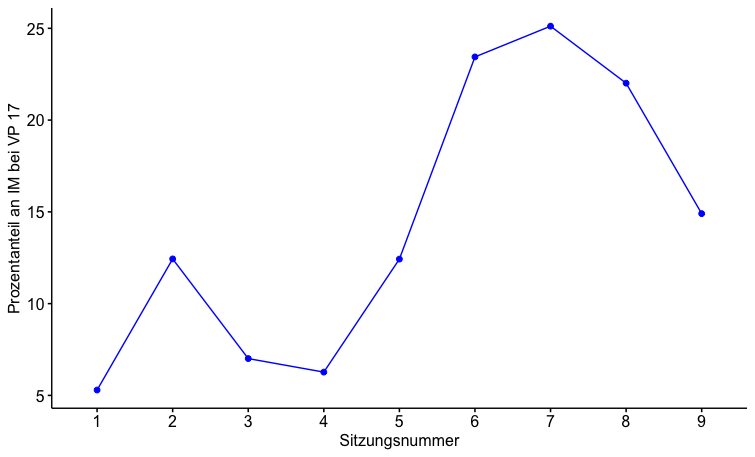

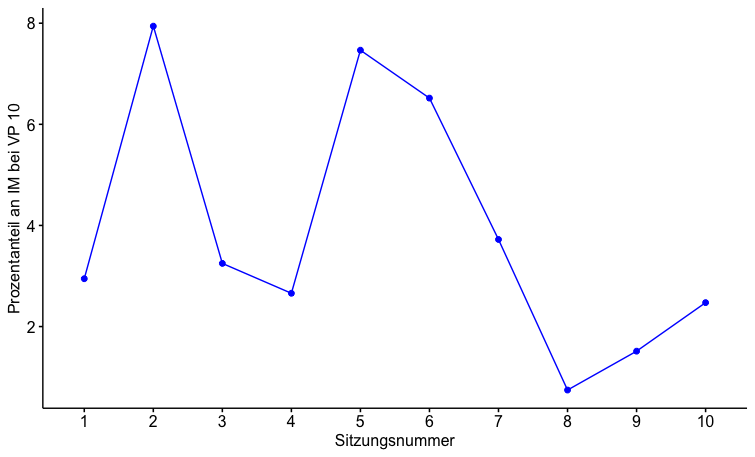
Supplementary material II: Individual Courses of Innovative Moments and depression.


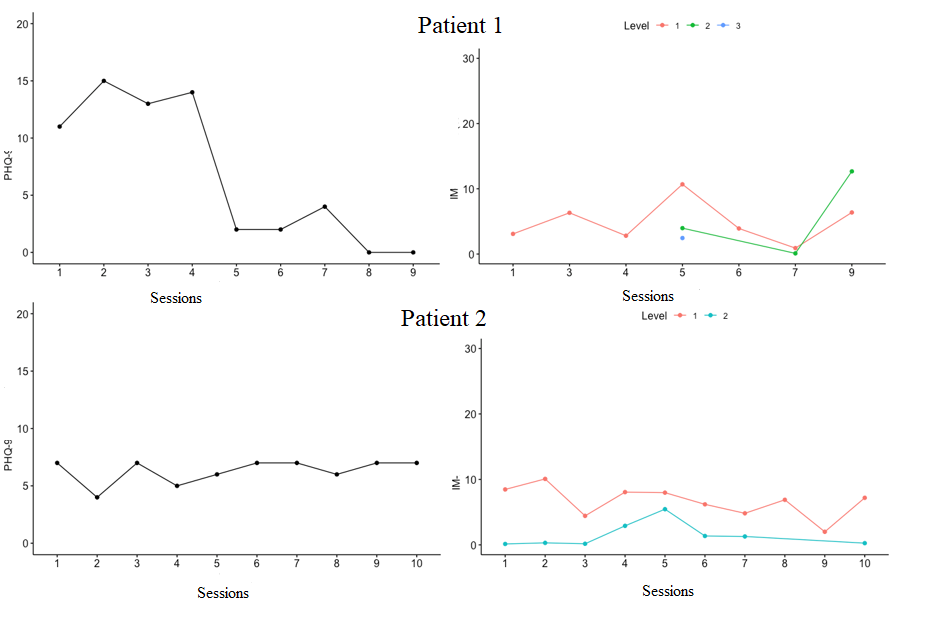


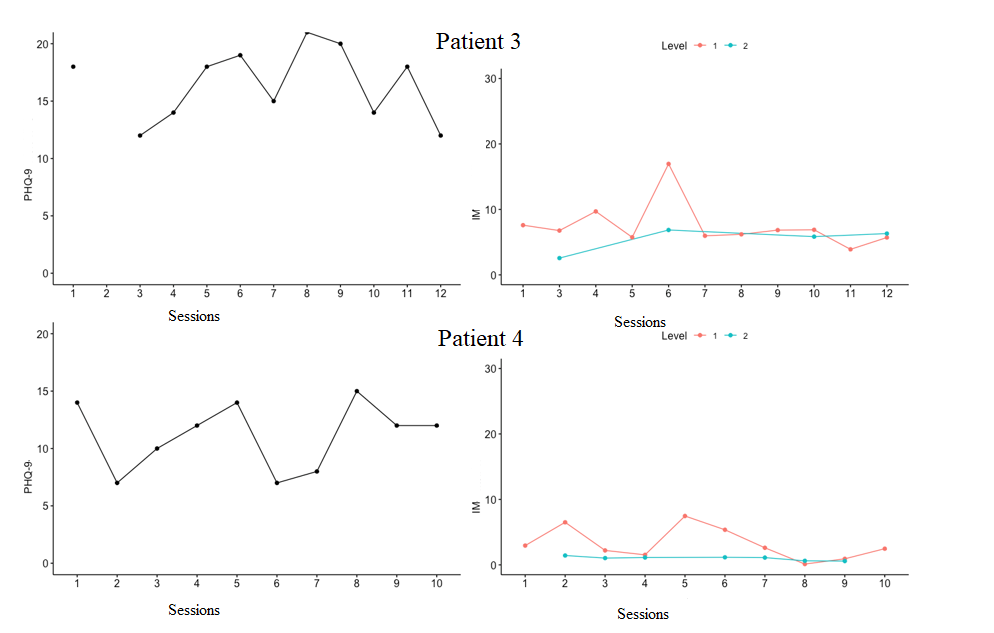


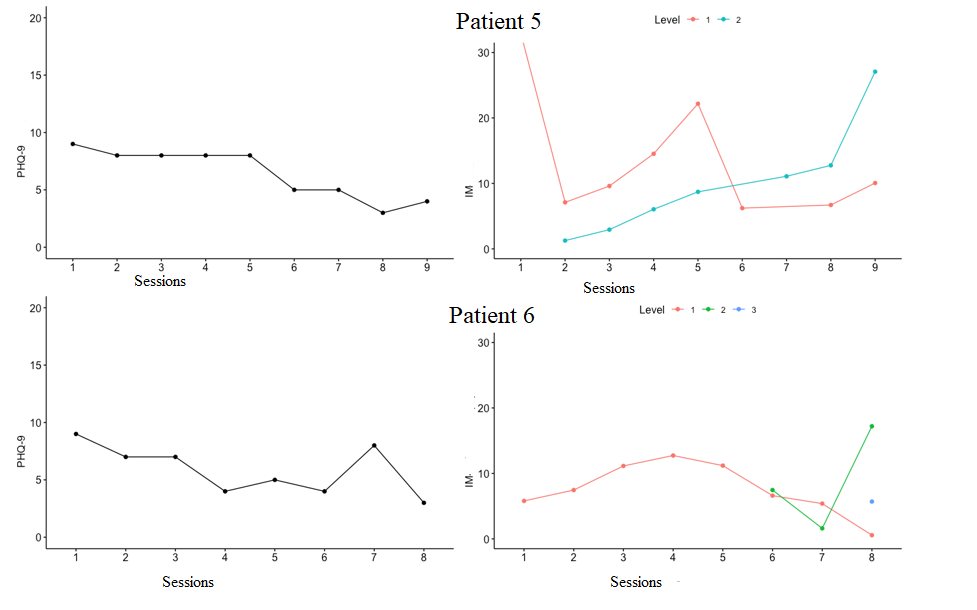


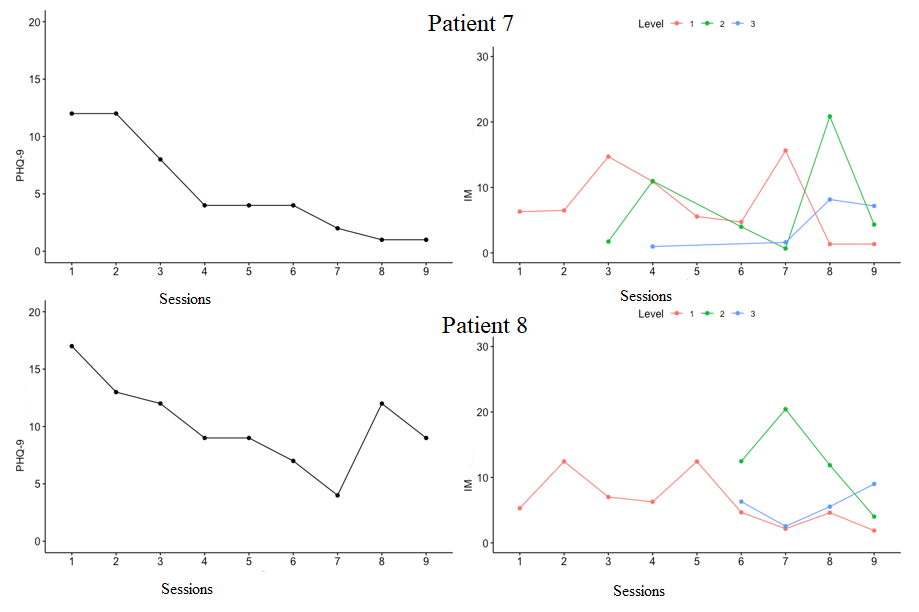


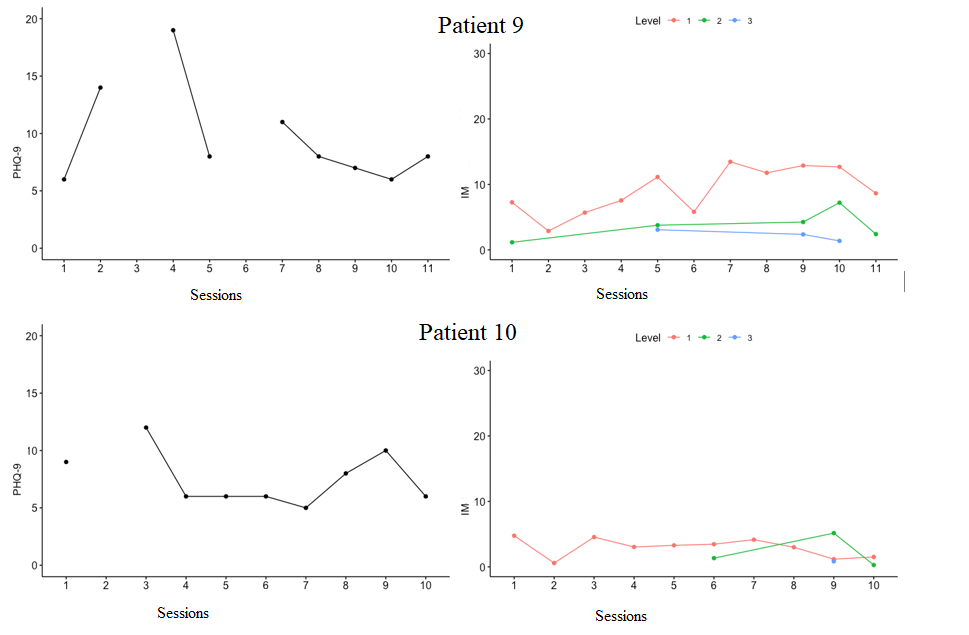


Note : IM = Innovative Moment, PHQ-9 = Patient Health Questionnaire (Kroenke et al., 2001).

Supplementary material III : Model fit and test statistics of single regressions

|  | *F*-statistic | 𝛽 | *p*-value | *R^2^* |
| --- | --- | --- | --- | --- |
| Total amount of IM | 0.73 | - 0.25 | .42 | .08 |
| Level 1 IM | 0.01 | - 0.06 | .91 | .0015 |
| Level 2 IM | 2.28 | - 0.88 | .17 | .2216 |
| Level 3 IM | 2.65 | - 2.35 | .14 | .2487 |

*Note*. IM = Innovative Moments
